# Automating approximate Bayesian computation by local linear regression

**DOI:** 10.1186/1471-2156-10-35

**Published:** 2009-07-07

**Authors:** Kevin R Thornton

**Affiliations:** 1 Department of Ecology and Evolutionary Biology, University of California Irvine, Irvine, CA, USA

## Abstract

**Background:**

In several biological contexts, parameter inference often relies on computationally-intensive techniques. "Approximate Bayesian Computation", or ABC, methods based on summary statistics have become increasingly popular. A particular flavor of ABC based on using a linear regression to approximate the posterior distribution of the parameters, conditional on the summary statistics, is computationally appealing, yet no standalone tool exists to automate the procedure. Here, I describe a program to implement the method.

**Results:**

The software package ABCreg implements the local linear-regression approach to ABC. The advantages are: 1. The code is standalone, and fully-documented. 2. The program will automatically process multiple data sets, and create unique output files for each (which may be processed immediately in R), facilitating the testing of inference procedures on simulated data, or the analysis of multiple data sets. 3. The program implements two different transformation methods for the regression step. 4. Analysis options are controlled on the command line by the user, and the program is designed to output warnings for cases where the regression fails. 5. The program does not depend on any particular simulation machinery (coalescent, forward-time, etc.), and therefore is a general tool for processing the results from any simulation. 6. The code is open-source, and modular.

Examples of applying the software to empirical data from *Drosophila melanogaster*, and testing the procedure on simulated data, are shown.

**Conclusion:**

In practice, the ABCreg simplifies implementing ABC based on local-linear regression.

## Background

In many biological applications, parameter inference for models of interest from data is computationally challenging. Ideally, one would like to infer parameters using either maximum likelihood or Bayesian approaches which explicitly calculate the likelihood of the data given the parameters. While such likelihoods can be calculated for data from non-recombining regions [1,2] and for data where all sites are independent [3,4], full-likelihood methods are not currently feasible for many models of interest (complex demography with recombination, for example). Therefore, approximations are desirable.

In the last several years, approximate methods based on summary statistics have gained in popularity. These methods come in several flavors:

1. Simulate a grid over the parameter space in order to calculate the likelihood of the observed summaries, given parameters [5,6]. The maximum-likelihood estimate is the point on the grid that maximizes the likelihood of the observed summary statistics.

2. The maximum-likelihood algorithm can be modified to perform Bayesian inference by simulating parameters from prior distributions, calculating summary statistics, and accepting the parameters if they are "close enough" to the observed [7,8]. The method runs until the desired number of acceptances are obtained, and can be extremely time-consuming. I refer to this approach as rejection sampling, and it has been applied in several contexts [9-11].

3. Decide ahead of time how many random draws to take from a prior distribution, then accept the fraction of draws which generate summary statistics closest to the data, according to some distance metric. This is the rejection-sampling approach of [12], and differs from the approach of [7-11] in that a *finite number of simulations are performed from the prior *instead of repeatedly simulating from the prior until a desired number of acceptances are recorded.

4. Take the parameters accepted from Method 3, and regress those acceptances onto the distance between the simulated and observed summary statistics [12].

The latter three methods are all forms of "Approximate Bayesian Computation" (ABC), a term which generally applies to inference problems using summary statistics instead of explicit calculations of likelihoods. The three Bayesian schemes described above are the simplest form of ABC, and the approach has been extended to use Markov Chain Monte Carlo techniques to explore the parameter space [13] and sequential Monte Carlo [14]. Further developments include formalizing methods for choosing summary statistics [15] and methods for model selection [16]. In this paper, I will use "regression ABC" to refer to Method 4, the regression approach of [12]. The main appeal of regression ABC is speed, overcoming a major limitation of rejection-sampling, which is often too slow to feasibly evaluate the performance of the estimator (due to requiring high rejection rates in order to obtain reasonable estimates [8,11]). In general, the regression ABC method has several appealing features, including simplicity of implementation, speed, and flexibility. The flexibility is a key issue, as it allows one to rapidly explore how many, and which, summary statistics to use, which is an important issue, as subtle choices can lead to surprising biases in estimation [17].

Currently, many tools are available for the rapid development and testing of summary-statistic based approaches to inference, including rapid coalescent simulations for both neutral models [18] and simple models of selection [9,19,20], software to calculate summary statistics from simulation output [21], and open-source statistical packages such as R[22]. Currently, the only software package available to implement the regression algorithm of [12] is implemented in the R language, and is available from . The purpose of this paper is to describe a software package which automates the linear regression portion of regression ABC analyses in a fast and flexible way, with user-friendly features simplifying automation. The results from the current code have been validated against independent R implementations, and the "ABCreg" package is fully documented for use by non-programmers.

## Implementation

The software package is called ABCreg, and is distributed as source code from the author's web site (see below). The code compiles to generate a single binary, reg, which automates all of the regression computations. The code was written in the C++ programming language [23], and the linear algebra calculations for the regression are performed using the GNU Scientific Library (GSL, ). The C and C++ languages are ideal for this task due to the speed of the compiled programs (often an order of magnitude faster than R). Although the regression-ABC step is less computationally-demanding than simulating from the prior distribution, it does not necessarily follow that the relative speed of the simulations is the limiting step in an analysis. In practice, one may spend considerable time evaluating the utility of different sets of summary statistics, running the regression-ABC portion of the analysis multiple times on a set of simulated data. It is therefore desirable to optimize the speed of the regression-ABC step as well as the speed of the simulations.

The algorithm implemented is identical to that of [12]. In brief, the reg program performs the following operations:

1. Transformation of the parameters simulated from the prior distribution. Currently, the program implements both the natural-log transformation used in [12] and the transformation proposed by [24]:



where *min *and *max *are the lower and upper bounds of the prior, respectively. The latter transformation assures that the posterior distribution is contained withing the bounds of the prior. The user may also opt to not transform the simulated values at all.

2. Normalisation of the observed summary statistics and summary statistics simulated from the prior

3. The rejection step based on accepting the closest *δ *of Euclidean distances between observed an simulated summary statistics. Here, *δ *specifies the tolerance for acceptance, and is the fraction of draws from the prior to accept, specified by the user on the command line.

4. The regression adjustment

5. Back-transformation of regression-adjusted parameter values and output to files. The program generates one output file per data set in the data file. File names are generated automatically, and the prefix of the file names is controlled by the user. The output files contain tab-delimited columns which are the regression-adjusted parameter values (*i.e*., the estimates of the posterior distribution), which are easily processed in R.

Use of the software requires two input files. The first file describes the data (either real or simulated), and contains a space-delimited list of the summary statistics. One can analyze multiple data sets by recording the summary statistics for each data set on a different line of the file. The second input file describes the results of simulating from the prior distribution on the model parameter(s). This "prior file" contains a space-delimited list of the parameters, and the corresponding summary statistics (in the same order as in the data file).

Additional features include a complete debugging mode, which helps identify cases where the linear regression may fail. In practice, the analysis of some data sets may return non-finite parameter values. Often, this is due to the predicted mean value of the regression being quite large, such that back-transformation (+/- the residuals from the regression) results in a value that cannot be represented on the machine. In debug mode, such cases immediately exit with an error. When not in debug mode, the program prints warnings to the screen.

## Results

In this section, I show results from applying the ABCreg software to the inference scheme of [11], who used rejection sampling (Method 2 above) to infer the parameters of a simple population bottleneck model from sequence data obtained from a European population sample of *Drosophila melanogaster*. This model has three parameters, *t*_*r*_, the time at which the population recovered from the bottleneck, *d*, the duration of the bottleneck, and *f*, the bottleneck severity. The parameters *t*_*r *_and *d *are scaled in units of 4*N*_0 _generations, where *N*_0 _is the effective population size at the present time, and *f *= *N*_*b*_/*N*_0_, the ratio of the bottlenecked size to the current size (0 <*f *≤ *N*_0_). See [11] for more details of the model. The data consist of 105 X-linked, non-coding loci surveyed by [25] and another ten from [10]. For each of these 115 non-coding fragments, sequence variation was surveyed in population samples from Zimbabwe, and the Netherlands. Thornton and Andolfatto used a two-step approach for the parameter inference. First, a relatively wide uniform prior was used in conjunction with a fairly liberal tolerance for acceptance. Then, the 1^*st *^and 99^*th *^quantiles of the resulting posterior distributions were used as the bounds on a new, uniform prior, and the acceptance criteria were made more strict. Three summary statistics were used: the variances across loci of nucleotide diversity (*π*, [26]), the number of haplotypes in the sample, and a summary of the site-frequency spectrum of mutations [27]. The rejection sampling scheme took two weeks to run on a large computer cluster.

I repeated the analysis using the local regression approach using the same data and uniform priors on parameters (see Table one of [11]). The analysis was done assuming that *ρ *= 4*N*_*e*_*r *(the population recombination rate) is equal to 10*θ *(see [11] for details), and the value of *θ *(the population mutation rate, see [28], p. 92) at each locus was obtained by the method of [29] using data from a Zimbabwe population sample. C++ code was written using the GSL and the coalescent routines in libsequence[21] to sample 5 × 10^6 ^draws from the prior distribution on the three parameters, and to record the resulting summary statistics. Simulating from the prior took 24 hours on four 2 gigahertz AMD Opteron processors. The tolerance was set such that 10^3 ^acceptances were recorded for the regression. The model has three parameters, and three summary statistics are used. Once the simulations from the prior distribution are complete, the entire ABC analysis was performed with one command:

**reg -P 3 -S 3 -p prior -d data -b data -t 0.0002 -T**,

where the arguments specify the number of parameters (-P), number of summary statistics (-S), names of files containing the prior (-p) and data (-d), the prefix of the output file names (-b), the tolerance (-t), and -T specifies the transformation described in [24]. The reg command takes seconds to run on a desktop CPU. Thus, the entire inference procedure took roughly 1 day using 4 CPU, compared to the original analysis based on rejection sampling, which took many CPU-months [11]

**Figure 1:**
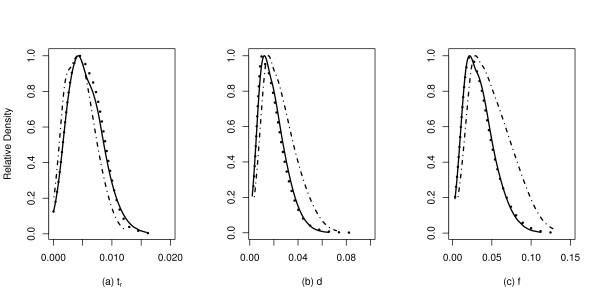
**Estimation of bottleneck parameters for European populations of *Drosophila melanogaster***. The data analyzed are described in [11]. The regression ABC was performed with both tangent [24] and logarithmic transformations [12]. In each panel, the solid line is the approximate posterior distribution obtained using the regression-ABC algorithm and the natural-log transformation, the dotted line is the result of regression-ABC using the transformation from [24], and the dot-dashed line are the rejection sampling results from [11]. The parameters are (a), *t*_*r *_the recovery time from the bottleneck, in units of 4*N*_*e *_generations, (b) *d*, the duration of the bottleneck in units of 4*N*_*e *_generations, and (c) *f*, the severity of the bottleneck, which is the ratio of the bottlenecked population size to the pre-bottleneck population size.

Figure 1 shows the comparison of the output from reg to the rejection sampling results of [11]. The regression and rejection approaches give very similar results for the time the population recovered from the bottleneck (Figure 1a), but the regression approach gives posterior distributions that are slightly left-shifted and have smaller variances, relative to the rejection sampling for both the duration (Figure 1b) and severity (Figure 1c) of the bottleneck. The major difference between the methods, however, is the total computation time required-approximately one day on four processors for the regression approach, compared to 14 days on 100 processors for the rejection-sampling approach.

**Figure 2:**
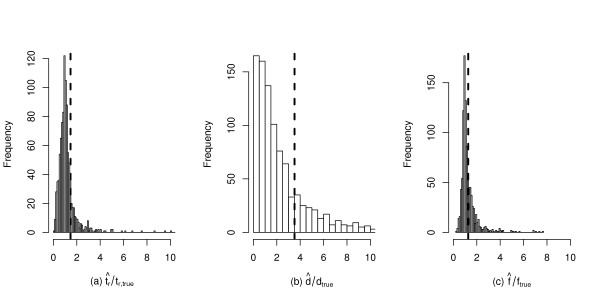
**Performance of the regression ABC estimator of bottleneck parameters**. Parameters were estimated from the modes of posterior distributions from one thousand random samples from the prior model used for inference in Figure 1. Because each data set is a random sample from a distribution of parameters, the distribution of each estimator is divided by the true value, such that the distribution of an unbiased estimator would have a mean of one. A vertical line is placed at the mean of each distribution. The parameters are the same as in Figure 1. As in Figure 1, the tolerance was set to accept 10^3 ^draws from the prior, and the tangent transformation was used prior to regression [24].

Because the method is quite rapid, the performance of the estimator is easily evaluated. Figure 2 shows the result of testing the estimator on 10^3 ^random samples from the prior model used for the inference in Figure 1. The properties of the estimator are qualitatively similar to those reported in [11], but were much faster to obtain (about 20 minutes of computation time on a desktop computer, compared to 160 minutes when the procedure is scripted in R).

## Conclusion

The linear regression approach to ABC analysis [12] is a fast and flexible method of performing parameter inference from population-genetic data. The software described here facilitates such analyses in a flexible way, and is designed to interact seamlessly with widely-available tools for population-genetic simulation and statistical analysis.

## Availability and requirements

The source code is distributed under the terms of the GNU public license and is available from the software section of the author's web site . Documentation is also available online, as is a shell script containing a complete example. The software was developed and tested on Linux and Apple's OS X platforms, using the gcc compiler suite . In order to compile and use the software, the GNU C++ compiler (g^++^) is needed, and GSL must be installed on the system. The GSL is readily available as a pre-compiled package on many Unix-like systems, and is easily installable from source code on any system with a C compiler.

## Authors' contributions

The author implemented and tested the code, and wrote the paper.
